# Tea consumption and risk of lung diseases: a two‑sample Mendelian randomization study

**DOI:** 10.1186/s12890-023-02762-4

**Published:** 2023-11-22

**Authors:** Linjie Chen, Yaru Deng, Tiexu Wang, Xinyu Lin, Lukun Zheng, Xiaohong Chen, Tongsheng Chen

**Affiliations:** 1 Institute of Respiratory Diseases, Xiamen Medical College, Xiamen, Fujian Province 361023 P.R. China; 2 Fujian Provincial Key Laboratory of Functional and Clinical Translational Medicine, Xiamen Medical College, Xiamen, Fujian Province 361023 P.R. China; 3 Department of Physiology, Xiamen Medical College, Xiamen, Fujian Province 361023 P.R. China; 4 Department of Clinical Medicine, Xiamen Medical College, Xiamen, Fujian Province 361023 P.R. China

**Keywords:** Tea consumption, Lung diseases, Mendelian randomization, COPD, IPF, Lung cancer

## Abstract

**Background:**

Numerous studies have reported the association between tea intake and lung diseases. However, the probable relationship between tea consumption on lung diseases still remain controversial and it is unclear whether these findings are due to reverse causality or confounding factor.

**Methods:**

In order to systematically investigate the causal connection between tea intake on respiratory system disorders, we employed a two-sample Mendelian randomized (MR) study. Genetic instruments for tea intake were identified from a genome-wide association study (GWAS) involving 447,385 individuals. Data on lung diseases were collected from a variety of publicly available genome-wide association studies. The main method used for MR analysis is the inverse variance weighting (IVW) method. To ensure the accuracy of the findings, further sensitivity analysis was conducted.

**Results:**

The IVW method in our MR analysis revealed no evidence to support a causal relationship between tea intake and lung diseases (IPF: OR = 0.997, 95% CI = 0.994-1.000, *p* = 0.065; Lung cancer: OR = 1.003, 95% CI = 0.998–1.008, *P* = 0.261; COPD: OR = 1.001, 95% CI = 0.993–1.006, *p* = 0.552; acute bronchitis: OR = 0.919, 95% CI = 0.536–1.576, *p* = 0.759; tuberculosis: OR = 1.002, 95% CI = 0.998–1.008, *p* = 0.301; pneumonia: OR = 0.789, 95% CI = 0.583–1.068, *p* = 0.125). The reliability of the results was further demonstrated by four additional MR analysis techniques and additional sensitivity testing.

**Conclusion:**

We found no evidence of a link between tea intake on lung diseases in our MR results based on genetic information.

**Supplementary Information:**

The online version contains supplementary material available at 10.1186/s12890-023-02762-4.

## Introduction

Lung diseases pose a serious threat to our society in terms of both the economy and public health. Recent published data revealed that 3,914,196 fatalities in 2017 were attributed to respiratory disorders, a rise of 18% since 1990, making them the third highest cause of death overall [1]. Globally, chronic respiratory diseases (CRD) had a global prevalence of 454.6 million cases and resulted in 4 million deaths. Among the 454.6 million patients with CRD, 212.3 million (204-225.1) had COPD, which is the primary cause of deaths related to CRD, accounting for 3.3 million (29 − 3.6) deaths [2]. The predominant causes of death in COPD, in order of prevalence, are concurrent cardiovascular diseases, cancer, infections, and chronic lung diseases [3]. The total direct (medical) and indirect (lost productivity) costs of COPD, lung cancer, tuberculosis, and other diseases are projected to be at least 96 billion euros per year in 28 EU nations, according to the European Lung White Book published in 2013 [4]. Lung diseases such as idiopathic pulmonary fibrosis (IPF), lung cancer, chronic obstructive pulmonary disease(COPD), pneumonia, acute bronchitis, and tuberculosis are at least partially associated with inflammation and inflammation-related oxidative stress [5, 6]. Over the past several years, many countries have been striving to prevent or treat lung disease, but the present outcomes of these efforts have not yet attained a desirable level. Therefore, ongoing and effective prevention measures and strategies remain key factors in managing lung disease.

Tea, the world’s second popular beverage, contains a number of bioactive substances, including polyphenols, flavonoids, and theanine [7, 8]. Numerous studies have shown drinking tea have many health benefits, such as a decreased risk of diabetes [9, 10], cardiovascular disease [11, 12], and several tumor diseases [13, 14]. However, the association between drinking tea and respiratory diseases remain controversial. One study showed that the catechins present in green tea extract significantly reduced the degree of fibrosis in animal models of radiation-induced pulmonary fibrosis [15]. Another study, however, showed that green tea extract impaired the clinical treatment efficacy of pulmonary fibrosis [16]. Several studies demonstrated the protective effects of green tea extract against emphysema in individuals with COPD [17, 18]. A meta-analysis study showed that drinking black and green tea linked with the risk of getting lung cancer [19], which was in contradiction with another study [20]. In fact, traditional epidemiological studies can be affected by potential confounders and reverse causality, which can lead to overestimation or underestimation of the causal link between causes and outcomes. It remains unclear whether the observed association between tea consumption and the risk of lung diseases is causal.

Mendelian randomization (MR) is a novel method for explaining observation bias [21]. The method integrates pooled data from genome-wide association studies (GWAS) and uses single nucleotide polymorphisms (SNPs) as instrumental variables to infer causal relationships between exposure and outcome [22]. Because of the random assignment of genetic variants during meiosis, it is possible to simulate natural randomized controlled trials, thus minimizing the interference of confounding factors and reverse causality in traditional epidemiology, and avoiding the difficulties and ethical issues associated with the implementation of randomized controlled trials [23, 24]. In order to investigate the causal link between tea consumption and lung diseases, we performed an MR analysis.

## Materials and methods

### Study design

**Fig. 1:**
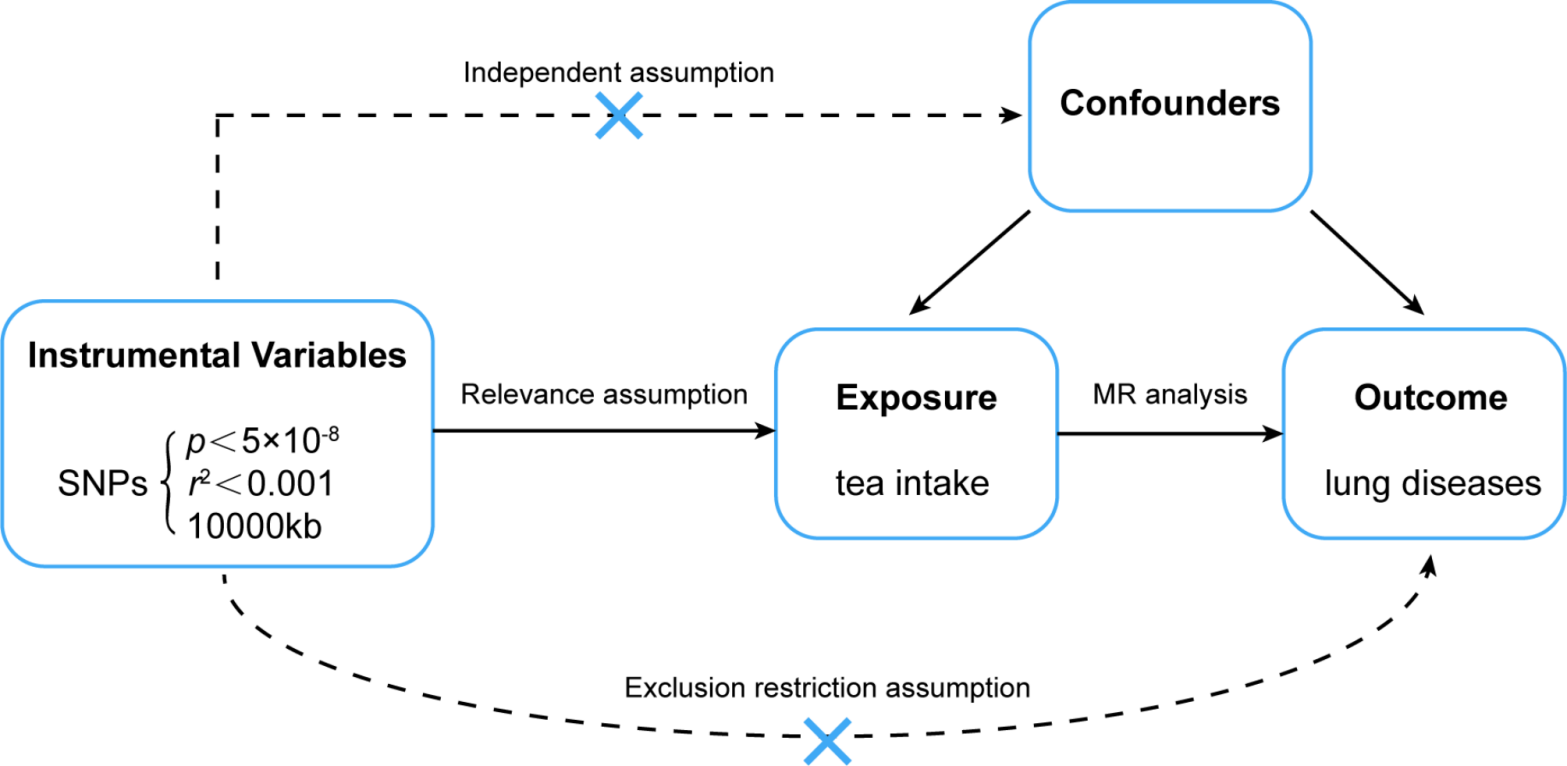
Design of Mendelian randomization study of tea intake and lung diseases The instrumental variable in this Mendelian randomization study was based on the hypothesis that it was related to tea intake but not to confounding variables, and that it only impacted the risk of six lung diseases through tea intake

We conducted a two-sample Mendelian randomization analysis to investigate the causal association between tea intake and lung diseases, using summary statistics from GWAS datasets. The exposure and outcome variables in our analysis were derived from separate GWAS datasets. We performed sensitivity analyses using different MR methods with varying model assumptions. Our study was based on three fundamental assumptions: first, a strong correlation must exist between the instrumental variable and the exposure factor; second, the instrumental variable must not be associated with any potential confounders; and third, the instrumental variable can only affect the outcome through the exposure variable [25]. The present concept of MR research is schematically displayed in Fig. 1.

### Data source

A large GWAS involving 447,485 samples of European ancestry was conducted by the MRC-IEU consortium, and the results discovered SNPs linked to tea consumption. This genome-wide association study (GWAS) was adjusted for various factors, including sex, genotyping arrays, and other variables. A questionnaire that asked participants how many cups of tea (both black and green tea) they typically drank each day was used to determine the participants’ habitual tea drinking behavior. According to the survey results, the mean value of tea intake was 3.51 cups per day, with a standard deviation of 2.85 cups. Additional information can be obtained in the UKBB release 2 data (https://biobank.ctsu.ox.ac.uk/crystal/field.cgi?id=1488). Data on six lung diseases were collected from multiple sources, including the MRC-IEU, the Neale Lab, and the FinnGen consortium. For these exposures, detailed information on the data sources for instrumental variables can be found in Supplementary Table 1. Of particular significance, all of the individuals are of European ancestry.

### Selection of instrumental SNPs

We applied several measures to assure the quality of valid instrumental SNPs for our analysis (Fig. 1). First, we selected SNPs that showed a genome-wide significant association (*p* < 5E-08) with tea intake as instrumental variables (IVs). Second, we employed pairwise-linkage disequilibrium (LD) clumping to ensure the independence of all instrumental SNPs used in our study (clumping distance = 10,000 kb, *r*²< 0.001). Third, we utilized the F-statistic to quantify the intensity of genetic variation and excluded SNPs with an F-statistic less than 10, which indicated that the genetic variation in these SNPs was relatively weak and did not meet the criteria for our study [26]. Fourth, we excluded SNPs with minor allele frequencies (MAF) below 0.01. Fifth, To ensure that the effect of SNPs on exposure is attributed to the same allele as the effect on the outcome, palindromic SNPs with intermediate allele frequencies were excluded from the analysis [27]. Sixth, following the harmonization process, we conducted an assessment of the instrumental variables to identify any strong correlations with the outcome (*p* < 5E-08). If a significant association was found, those instrumental factors were removed from further analysis. Seventh, using Phenoscanner, a website that offers comprehensive data on the relationship between genotype and phenotype, to perform checks and then remove SNPs associated with confounding factors. Overall, the aforementioned initial three steps satisfy the assumption of relevance, while steps 3–6 fulfill the exclusion restriction assumption and indicate the characteristics of the selected instrumental variables in the outcome. The independence assumption is upheld by using Phenoscanner to detect SNPs connected to confounding variables. The step of examining SNPs related to confounding factors using Phenoscanner satisfies the independence assumption. After applying the aforementioned criteria, a final set of SNPs suitable for further analysis was obtained.

### Statistic analysis

The key analysis in this MR study was conducted using the inverse variance weighted (IVW) method [28]. The approach employs a weighted regression involving multiple genetic variants to assess causal effects. Each individual variant’s effect size is given a weighting during this step to account for how much it contributes to the total effect, but this assumes that there are no invalid instrumental variables [29]. Moreover, MR-Egger, weighted median, simple mode, and weighted mode were employed as supplementary methods to IVW [30]. Similar to IVW, the MR Egger method is a weighted regression of SNP results from SNP exposure associations, but unlike the IVW method, the intercept is not constrained to zero. The slope of the MR-Egger method gives an unbiased estimate even if all instruments are invalidated [31, 32]. The weighted median method combines data from multiple instrumental variables into a single causal estimate, which provides an accurate estimate when more than 50% of the weights are from valid instrumental variables and has a superior finite sample type 1 error rate compared to IVW [33, 34]. The weighted mode method estimates the causal effect by clustering SNPs into subsets and focusing on the subset with the highest number of SNPs [35, 36]. The simple mode offers robustness against pleiotropy, although it lacks as much power as IVW [37]. While IVW can provide the most accurate results when all instrumental variables used are valid SNPs, the other four methods have their own advantages and applicability in different situations. Therefore, if the results of the five methods are consistent, it can enhance the robustness of the findings.

To verify the validity of our conclusions, we conducted several tests to evaluate heterogeneity and horizontal pleiotropy. Initially, we used Cochran’s Q statistic to assess the heterogeneity of the SNPs [38]. We then examined horizontal pleiotropy using both MR pleiotropy residual sum and outlier (MR-PRESSO) analysis and MR-Egger intercept [39, 40]. MR-PRESSO was not only used to examine horizontal pleiotropy but was also utilized to detect and correct potential outliers in the instrumental variable analysis. We assessed the outcome to be untrustworthy if the *p*-value of the MR-Egger intercept was less than 0.05, and we assumed that the instrumental variable was strongly influenced by horizontal pleiotropy. To determine if a single SNP influences the causal link between tea consumption and lung illness, leave-one-out analysis was used [41].

All data analyses were conducted in R (version 4.3.0) using the R packages “TwoSampleMR” and “MR-PRESSO”. A value of *p* < 0.05 was chosen as the significance criterion for this MR analysis.

## Results

### SNPs associated with tea intake

We screened SNPs associated with tea intake and obtained a total of 41 SNPs (all SNPs had *p* values less than 5E-08 and *r*^2^ values under 0.001). The *F*-statistics of these SNPs were all greater than the conventional threshold of 10, indicating that the instrument bias was weak in our MR study and could not significantly affect the estimation of causal effects (Supplementary Table 2). During the screening process, we removed SNPs associated with lung diseases and its related confounders (rs2478875, rs4410790, rs2472297, rs9937354), and Palindromic structure SNPs (rs11164870, rs132904, rs1453548, rs2273447, rs2783129, rs56348300, rs713598, rs9302428), and SNPs that were not available in the outcome dataset. We give comprehensive information about all relevant SNPs, as shown in Supplementary Tables 3–8.

### Association between tea and lung disease

**Fig. 2:**
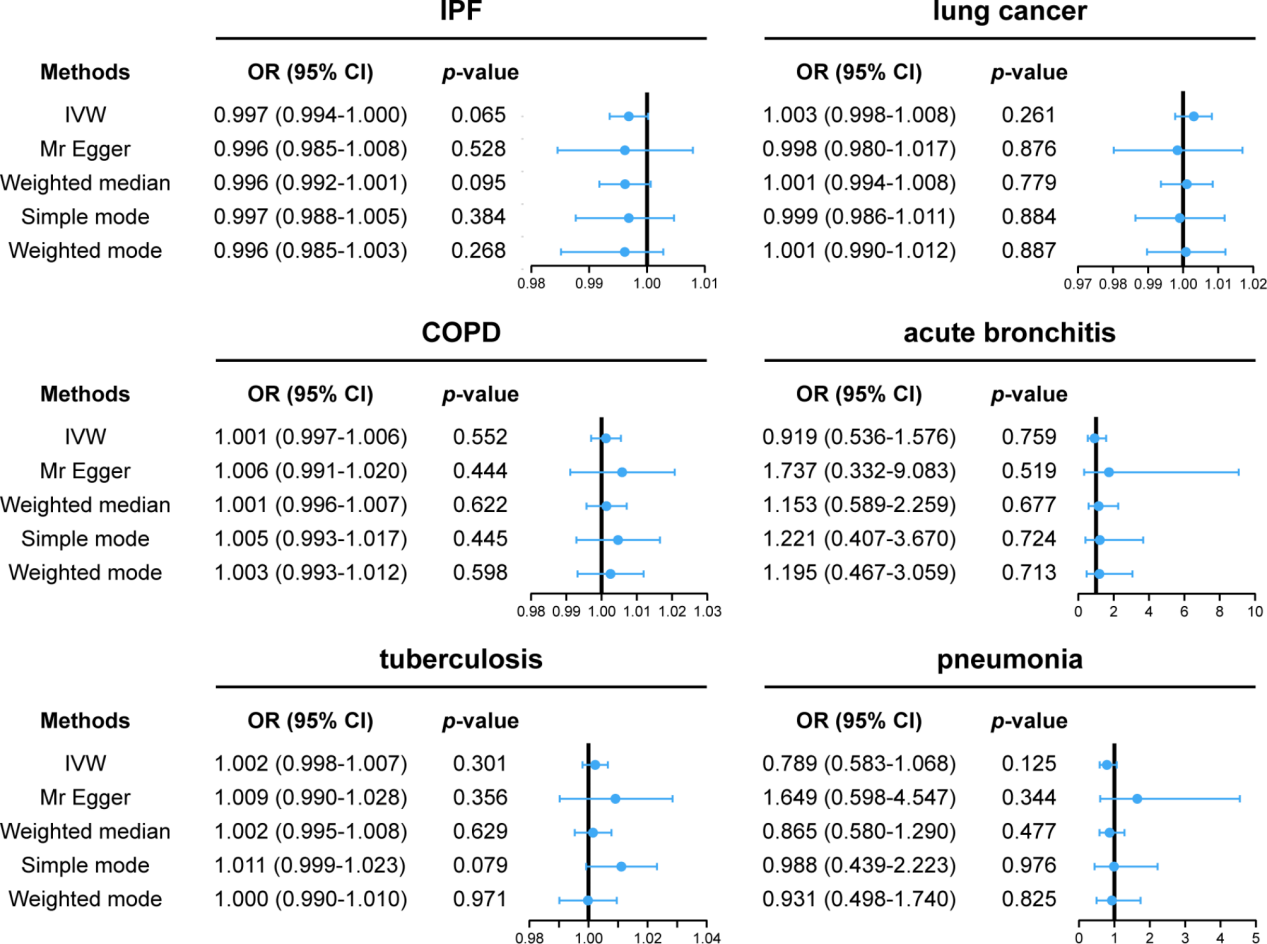
Forest plot showed the causal association between tea intake and lung disease. (**A**) IPF; (**B**) lung cancer; (**C**) COPD; (**D**) acute bronchitis; (**E**) tuberculosis; (**F**) pneumonia. OR, odds ratio; 95% CI, 95% confidence interval; IPF, idiopathic pulmonary fibrosis; COPD, chronic obstructive pulmonary diseases

The results of the IVW analysis revealed that a genetically predicted change in tea intake per unit SD (SD: 2.85 cups/day) was not causally associated with a decreased risk of six common lung disease (IPF: OR = 0.997, 95% CI = 0.994-1.000, *p* = 0.065; Lung cancer: OR = 1.003, 95% CI = 0.998–1.008, *p* = 0.261; COPD: OR = 1.000, 95% CI = 0.995–1.005, *p* = 0.934; acute bronchitis: OR = 0.919, 95% CI = 0.536–1.576, *p* = 0.759; tuberculosis: OR = 1.002, 95% CI = 0.998–1.008, *p* = 0.301; pneumonia: OR = 0.789, 95% CI = 0.583–1.068, *p* = 0.125) (Fig. 2). Similar outcomes were basically achieved by the complementary four methods: MR-Egger, weighted median, weighted mode, and simple mode. An outlier SNP (rs7757102) was found in the MR-PRESSO test when the outcome variable was COPD. In order to avoid the bias caused by it, we removed it and performed MR analysis again. MR analysis demonstrated no causal relationship between tea intake and COPD (COPD: OR = 1.001, 95% CI = 0.993–1.006, *p* = 0.552). For all respiratory diseases, the *p*-values for the Cochran’s Q statistic and the MR-Egger were larger than 0.05, showing that there was no significant horizontal pleiotropy and heterogeneity in the analysis results (Table 1).

**Table 1:** The result of pleiotropy and heterogeneity test

outcome	Pleiotropy *p*-value		Heterogeneity *p*-value	
	MR-Egger	MR-PRESSO	MR-Egger	IVW
IPF	0.906	0.177	0.158	0.191
Lung cancer	0.610	0.303	0.450	0.493
COPD	0.532	0.162	0.170	0.190
Acute bronchitis	0.432	0.150	0.068	0.072
Tuberculosis	0.475	0.523	0.285	0.307
Pneumonia	0.150	0.087	0.246	0.190

### Visualization of sensitivity analysis

**Fig. 3:**
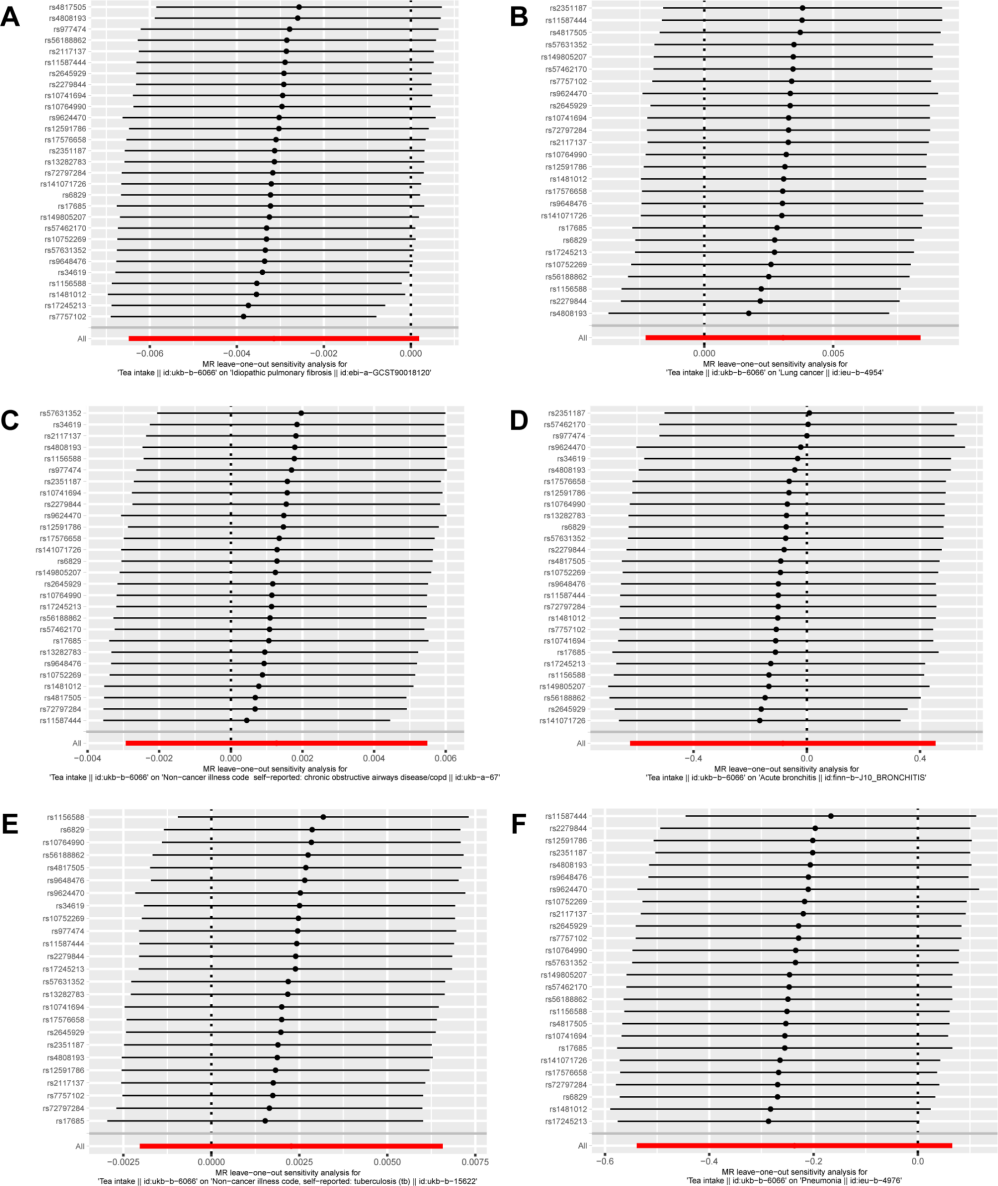
MR leave-one-out sensitivity analysis of tea intake on lung disease. Circles indicate the results of MR analysis of remaining SNPs on tea intake on lung disease after omitting each SNP in turn. Bars indicate CI. (**A**) IPF; (**B**) lung cancer; (**C**) COPD; (**D**) acute bronchitis; (**E**) tuberculosis; (**F**) pneumonia

**Fig. 4:**
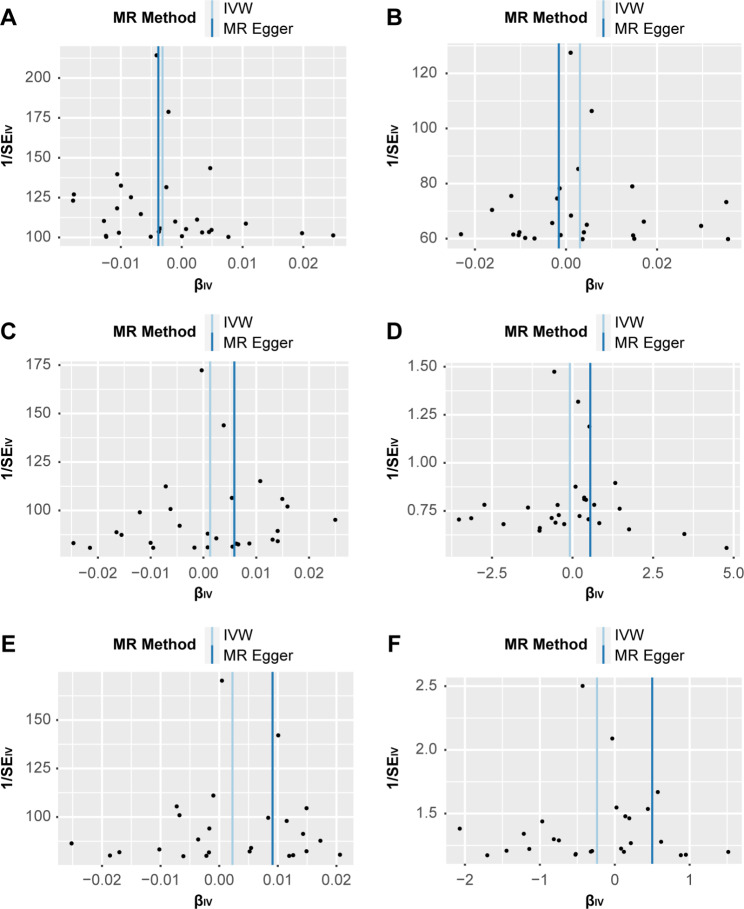
Estimating heterogeneity using funnel plots of individual causal relationships between tea intake and lung disease. (**A**) IPF; (**B**) lung cancer; (**C**) COPD; (**D**) acute bronchitis; (**E**) tuberculosis; (**F**) pneumonia

We conducted separate leave-one-out analyses for each outcome. The results consistently showed that none of the SNPs were significantly correlated with the non-causal relationship between tea intake and lung diseases (Fig. 3), and the symmetry of the funnel plot ruled out the possible influence of heterogeneity on our estimates throughout the estimation (Fig. 4).

## Discussion

In this study, the Mendelian randomization analyses were performed using a large sample GWAS database to determine the association between tea consumption and six common lung diseases. Our MR research found no evidence of a link between drinking tea and any of the six prevalent lung disease: IPF, lung cancer, COPD, tuberculosis, pneumonia, and acute bronchitis.

In the last decade, many epidemiologic studies have examined the relationship between tea consumption and respiratory diseases. However, to this day it has not been possible to draw definitive conclusions. For example, a study by Vu Thanh-Huyen T et al. showed a favorable correlation between tea and the incidence of pneumonia [42]. A large cohort study involving 19,079 men and 21,493 women showed that tea consumption was linked to a decreased risk of death from pneumonia in Japanese women [43]. However, a hospital-based case-control study found no association between pneumonia and tea drinking [44], which is in accord with our findings. Similarly, human studies have shown that green tea catechins does not affect the activity of the CYPIA2, CYP2D6, CYP2C9 and CYP3A4 enzymes, but in vitro studies have shown that green tea extract binds bortezomib and lessens its activity [45].

In this Mendelian randomization study, we did not observe a protective effect of tea consumption against several common lung diseases, which contradicted the results of some previous observational studies. In comparing our findings with those of previous studies, it is important to note that there are several potential reasons for this discrepancy. First, observational studies cannot be completely devoid of residual confounding and reverse causality. For instance, experiments in rats have shown that EGCG, a major component of green tea, may alleviate lung injury by inhibiting oxidative stress [46]. In addition to its antioxidant effects, the presence of several substances in tea, such as polyphenols, flavonoids, theanine, and EGCG, which have immunomodulatory, anti-inflammatory, is all related to the risk of respiratory diseases [47, 48]. Although a number of components in tea have been suggested as potentially beneficial, the biological effects of various tea polyphenols, including EGCG, have not been well studied, their effectiveness is limited by their low oral bioavailability, and there is insufficient evidence to support the idea that consuming tea can target respiratory disorders in humans through these components. Second, measurements of long-term tea intake in observational studies may be inaccurate. As in retrospective case-control studies, recall bias is also an issue to be addressed [49]. Finally, in reality, a completely random distribution of habitual tea drinkers is difficult to achieve. It is affected to some extent by a number of variables, including age, individual dietary habits, and the prevalence of tea culture in the region [50]. The composition of tea is complex, and the content of compounds varies among different types of tea. The mechanisms of action of different components may also differ. For instance, compared to oolong tea and black tea, green tea has a higher concentration of flavonoids [51]. In addition to the aforementioned primary reasons, there are a number of unavoidable interfering factors, such as taking medication for lung disease while in the habit of drinking tea, and the effects of medications such as erlotinib or nintedanib can be affected by green tea [52]. All of these elements could have an impact on the findings of prior observational research.

There are several strengths to this study. First, we applied MR methods for the first time to investigate the causal link between tea drinking and six common lung disease, which largely circumvents the limitations of conventional observational studies such as environmental confounders, reverse causality, and insufficient sample size. Second, using data with a sufficient number of large sample cases greatly increases the reliability of our findings. Third, because the current analysis was restricted to people with European ancestry, population stratification is unlikely to have had an impact on our findings. Our research offers fresh proof that there is no link between drinking tea and the risk of lung diseases. Fourth, we conducted multiple sensitivity analyses to validate the absence of horizontal pleiotropy and heterogeneity interference in our study, this suggests that there is no evidence of an illegal independence assumption in our analysis and potential outliers were removed by MR-PRESSO. Hereby further strengthened the reliability and consistency of our findings. To some extent, our work may contribute to further understanding of the impact of tea consumption on lung diseases and its role in dietary management for patients with lung diseases. However, it is important to emphasize the need for caution in interpreting our results due to the inherent limitations of MR analyses and suggests that a wider range of evidence should be considered in the development of dietary guidelines.

It is crucial to note that this study still has several inherent limitations. First, the fact that the data used in this study were obtained from a European database and that all subjects included in this study were of European ancestry precludes the generalizability of our findings to other ethnic groups. Second, due to the limitation of the corresponding information in the database, it is impossible to assess the influence of factors such as the classification of tea subtypes and diseases in the results, and it is inability to perform stratified analysis or consider other factors that may affect the relationship. Third, there may be some degree of sample overlap since the exposures and some of the results are from the UK Biobank. Even though sample overlap would raise the possibility of false positives, the results of this study were all negative, so the impact of sample overlap was minimal. Fourth, the selection of genetic instruments is based on statistical methods rather than biological criteria, which may result in a lower genetic power for tea consumption and ultimately lessen the real-world importance of the analysis. Fifth, the interaction between genes and environmental exposures as well as epigenetic phenomena such as methylation and histone modifications are all plays a crucial role in lung diseases. Unfortunately, we were unable to assess the effects of these in our current MR analysis.

## Conclusion

In conclusion, this MR study did not find a causal relationship between tea drinking and six common lung disease, including IPF, COPD, lung cancer, pneumonia, acute bronchitis, and tuberculosis. Despite the lack of a causal relationship between six lung diseases and tea consumption found by our Mendelian randomization analysis, this does not mean that tea and lung health are unrelated. Tea contains various components such as EGCG that have potential therapeutic effects on lung diseases. However, due to factors like oral bioavailability, it is challenging to utilize these potential active ingredients for treatment. Therefore, further research is warranted to explore the effective targeted delivery of these potential active compounds within the human body, ultimately aiming to achieve preventive or therapeutic effects on lung diseases and further studies are needed to confirm the findings and explore potential mechanisms.

### Electronic supplementary material

Below is the link to the electronic supplementary material.

**Supplementary Material 1: Table S1**: Detailed information of the genome-wide association study (GWAS) used in this study. **Table S2**: Mendelian randomization analysis of tea intake. **Table S3**: Mendelian randomization analysis of tea intake and IPF. **Table S4**: Mendelian randomization analysis of tea intake and lung cancer. **Table S5**: Mendelian randomization analysis of tea intake and COPD. **Table S6**: Mendelian randomization analysis of tea intake and acute bronchitis. **Table S7**: Mendelian randomization analysis of tea intake and tuberculosis. **Table S8**: Mendelian randomization analysis of tea intake and pneumonia

## Data Availability

All of the datasets used in this study came from publicly accessible resources found at (https://gwas.mrcieu.ac.uk/).
