# Supplementary material for: Tea consumption and risk of lung diseases: a two‑sample Mendelian randomization study
Source: BMC Pulm Med. 2023 Nov 22;23:461. doi: 10.1186/s12890-023-02762-4 (PMC10664472; doi:10.1186/s12890-023-02762-4)
Supplement: Supplementary file 1 — Supplementary Material 1: Table S1: Detailed information of the genome-wide association study (GWAS) used in this study. Table S2: Mendelian randomization analysis of tea intake. Table S3: Mendelian randomization analysis of tea intake and IPF. Table S4: Mendelian randomization analysis of tea intake and lung cancer. Table S5: Mendelian randomization analysis of tea intake and COPD. Table S6: Mendelian randomization analysis of tea intake and acute bronchitis. Table S7: Mendelian randomization analysis of tea intake and tuberculosis. Table S8: Mendelian randomization analysis of tea intake and pneumonia [file 12890_2023_2762_MOESM1_ESM.docx]

**Supplementary tables**

**Table S1: Detailed information of the genome-wide association study (GWAS) used in this study**

| **Study/Consortium** | **Exposure/Outcome** | **Cases** | **Controls** | **Sample size** | **Population** | **First author(Year)** |
| --- | --- | --- | --- | --- | --- | --- |
| MRC-IEU | Tea intake | - | - | 447,485 | European | Ben Elsworth(2018) |
| NA | Idiopathic pulmonary fibrosis | 1,369 | 435,866 | 451,025 | European | Duckworth A(2020) |
| UK Biobank | Lung cancer | 2,671 | 372,016 | 374,687 | European | Burrows(2021) |
| Neale Lab | COPD | 1,179 | 335,980 | 337,159 | European | Neale(2017) |
| UK Biobank | Pneumonia | 22,567 | 463,917 | 486,484 | European | Hamilton F(2021) |
| MRC-IEU | Tuberculosis | 2,277 | 460,656 | 462,933 | European | Ben Elsworth(2021) |
| FinnGen biobank | Acute bronchitis | 7,338 | 208,689 | 216,027 | European | 2021 |

**Table S2: Mendelian randomization analysis of tea intake**

| SNP | Chr | position | A1 | A2 | EAF | Beta | SE | P-value | R2 | F value |
| --- | --- | --- | --- | --- | --- | --- | --- | --- | --- | --- |
| rs11587444 | 1 | 150722844 | G | A | 0.3935 | 1.40E-02 | 0.0022 | 1.00E-10 | 9.34E-05 | 41.7883 |
| rs11164870 | 1 | 93552187 | G | C | 0.6046 | -1.20E-02 | 0.0022 | 4.20E-08 | 6.71E-05 | 30.0367 |
| rs56188862 | 1 | 174189269 | C | T | 0.3875 | -1.58E-02 | 0.0022 | 4.30E-13 | 1.17E-04 | 52.4971 |
| rs1156588 | 2 | 58515375 | G | A | 0.2101 | -1.55E-02 | 0.0026 | 2.90E-09 | 7.87E-05 | 35.2410 |
| rs57462170 | 3 | 50239803 | A | G | 0.1088 | 1.92E-02 | 0.0034 | 1.90E-08 | 7.07E-05 | 31.6201 |
| rs2117137 | 3 | 89525505 | G | A | 0.4051 | 1.30E-02 | 0.0022 | 1.70E-09 | 8.12E-05 | 36.3380 |
| rs1481012 | 4 | 89039082 | G | A | 0.1122 | -2.62E-02 | 0.0034 | 5.30E-15 | 1.37E-04 | 61.1473 |
| rs34619 | 5 | 60465365 | A | G | 0.4309 | 1.17E-02 | 0.0021 | 4.30E-08 | 6.71E-05 | 30.0210 |
| rs72797284 | 5 | 152031650 | G | A | 0.2708 | -1.71E-02 | 0.0024 | 7.00E-13 | 1.15E-04 | 51.5579 |
| rs7757102 | 6 | 137222671 | G | A | 0.5554 | -1.18E-02 | 0.0021 | 3.10E-08 | 6.84E-05 | 30.6238 |
| rs2478875 | 6 | 51283110 | G | A | 0.2088 | 2.19E-02 | 0.0026 | 5.10E-17 | 1.57E-04 | 70.2991 |
| rs149805207 | 6 | 137095269 | G | A | 0.0085 | -7.19E-02 | 0.0126 | 1.10E-08 | 7.30E-05 | 32.6846 |
| rs4410790 | 7 | 17284577 | C | T | 0.6312 | 4.06E-02 | 0.0022 | 3.40E-76 | 7.62E-04 | 341.2682 |
| rs17685 | 7 | 75616105 | A | G | 0.2775 | 2.31E-02 | 0.0024 | 1.60E-22 | 2.13E-04 | 95.3635 |
| rs141071726 | 7 | 17558580 | A | G | 0.0267 | 4.07E-02 | 0.0068 | 2.20E-09 | 7.99E-05 | 35.7534 |
| rs9648476 | 7 | 39293033 | A | G | 0.6230 | 1.25E-02 | 0.0022 | 1.10E-08 | 7.31E-05 | 32.7219 |
| rs713598 | 7 | 141673345 | G | C | 0.4023 | 1.34E-02 | 0.0022 | 5.20E-10 | 8.62E-05 | 38.5897 |
| rs13282783 | 8 | 22088975 | T | C | 0.2859 | -1.36E-02 | 0.0024 | 7.90E-09 | 7.44E-05 | 33.2892 |
| rs56348300 | 9 | 7054124 | G | C | 0.1846 | 1.59E-02 | 0.0027 | 6.10E-09 | 7.55E-05 | 33.7985 |
| rs10764990 | 10 | 129152608 | A | G | 0.6072 | -1.22E-02 | 0.0022 | 1.90E-08 | 7.06E-05 | 31.5891 |
| rs10752269 | 10 | 12692902 | A | G | 0.5061 | -1.29E-02 | 0.0021 | 1.30E-09 | 8.24E-05 | 36.8781 |
| rs2351187 | 10 | 86850616 | A | G | 0.3189 | 1.29E-02 | 0.0023 | 1.60E-08 | 7.14E-05 | 31.9585 |
| rs17245213 | 11 | 1679769 | A | G | 0.2080 | -1.46E-02 | 0.0026 | 2.00E-08 | 7.04E-05 | 31.5207 |
| rs10741694 | 11 | 16286183 | C | T | 0.6279 | 1.50E-02 | 0.0022 | 7.90E-12 | 1.05E-04 | 46.7843 |

| **Table S2 (continued): Mendelian randomization analysis of tea intake** | | | | | | | | | | |
| --- | --- | --- | --- | --- | --- | --- | --- | --- | --- | --- |
| SNP | Chr | position | A1 | A2 | EAF | Beta | SE | P-value | R2 | F value |
| rs1453548 | 11 | 59192089 | A | T | 0.6649 | -1.33E-02 | 0.0022 | 3.00E-09 | 7.86E-05 | 35.1674 |
| rs977474 | 12 | 11284772 | T | C | 0.8337 | 2.18E-02 | 0.0029 | 2.40E-14 | 1.30E-04 | 58.1800 |
| rs2783129 | 13 | 80168720 | G | C | 0.4849 | -1.17E-02 | 0.0021 | 3.80E-08 | 6.76E-05 | 30.2541 |
| rs17576658 | 13 | 100272019 | A | G | 0.2471 | -1.35E-02 | 0.0025 | 4.10E-08 | 6.73E-05 | 30.1165 |
| rs6829 | 13 | 111531264 | T | C | 0.5962 | -1.19E-02 | 0.0022 | 3.70E-08 | 6.77E-05 | 30.2817 |
| rs2645929 | 13 | 56444529 | G | A | 0.8131 | -1.50E-02 | 0.0027 | 3.50E-08 | 6.80E-05 | 30.4238 |
| rs12591786 | 15 | 60902512 | T | C | 0.1588 | -1.84E-02 | 0.0029 | 3.70E-10 | 8.78E-05 | 39.2738 |
| rs2472297 | 15 | 75027880 | T | C | 0.2620 | 5.33E-02 | 0.0024 | 2.30E-109 | 1.10E-03 | 493.6434 |
| rs9937354 | 16 | 53799847 | A | G | 0.4241 | -1.41E-02 | 0.0021 | 4.90E-11 | 9.66E-05 | 43.2311 |
| rs9302428 | 16 | 24717600 | G | C | 0.6358 | 1.22E-02 | 0.0022 | 2.60E-08 | 6.92E-05 | 30.9484 |
| rs2279844 | 17 | 40819809 | A | G | 0.3793 | -1.20E-02 | 0.0022 | 4.00E-08 | 6.74E-05 | 30.1512 |
| rs4808193 | 19 | 19410622 | C | T | 0.3353 | 1.51E-02 | 0.0022 | 1.70E-11 | 1.01E-04 | 45.2403 |
| rs57631352 | 19 | 4338173 | G | A | 0.2969 | -1.31E-02 | 0.0023 | 1.70E-08 | 7.12E-05 | 31.8683 |
| rs2273447 | 20 | 62900120 | T | A | 0.2038 | 1.75E-02 | 0.0026 | 3.30E-11 | 9.83E-05 | 43.9904 |
| rs4817505 | 21 | 34343828 | C | T | 0.3900 | 1.51E-02 | 0.0022 | 4.20E-12 | 1.07E-04 | 48.0121 |
| rs132904 | 22 | 41798896 | C | G | 0.7787 | 1.66E-02 | 0.0026 | 7.80E-11 | 9.45E-05 | 42.2956 |
| rs9624470 | 22 | 24820268 | A | G | 0.5801 | 2.52E-02 | 0.0022 | 1.30E-31 | 3.06E-04 | 136.8390 |

Abbreviation: SNP, single nucleotide polymorphism; Chr, chromosome; EAF, effect allele frequency; SE, standard error.

**Table S3: Mendelian randomization analysis of tea intake and IPF**

| SNP | Chr | position | A1 | A2 | EAF | Beta | SE | P-value |
| --- | --- | --- | --- | --- | --- | --- | --- | --- |
| rs11587444 | 1 | 150722844 | G | A | 0.3928 | -1.49E-04 | 0.0001 | 0.2100 |
| rs13282783 | 8 | 22088975 | T | C | 0.2856 | 4.79E-05 | 0.0001 | 0.7100 |
| rs2351187 | 10 | 86850616 | A | G | 0.3198 | -4.85E-05 | 0.0001 | 0.7000 |
| rs34619 | 5 | 60465365 | A | G | 0.4312 | 8.99E-05 | 0.0001 | 0.4400 |
| rs12591786 | 15 | 60902512 | T | C | 0.1584 | 1.24E-04 | 0.0002 | 0.4400 |
| rs17576658 | 13 | 100272019 | A | G | 0.2468 | 6.86E-05 | 0.0001 | 0.6100 |
| rs57631352 | 19 | 4338173 | G | A | 0.2974 | -5.89E-05 | 0.0001 | 0.6400 |
| rs4817505 | 21 | 34343828 | C | T | 0.3919 | -2.67E-04 | 0.0001 | 0.0240 |
| rs9624470 | 22 | 24820268 | A | G | 0.5806 | -1.04E-04 | 0.0001 | 0.3800 |
| rs9648476 | 7 | 39293033 | A | G | 0.6230 | 6.13E-05 | 0.0001 | 0.6100 |
| rs17245213 | 11 | 1679769 | A | G | 0.2074 | -2.90E-04 | 0.0001 | 0.0420 |
| rs10764990 | 10 | 129152608 | A | G | 0.6063 | 1.26E-04 | 0.0001 | 0.2900 |
| rs17685 | 7 | 75616105 | A | G | 0.2777 | -5.02E-05 | 0.0001 | 0.7000 |
| rs1156588 | 2 | 58515375 | G | A | 0.2103 | -1.63E-04 | 0.0001 | 0.2500 |
| rs2117137 | 3 | 89525505 | G | A | 0.4053 | -1.66E-04 | 0.0001 | 0.1600 |
| rs57462170 | 3 | 50239803 | A | G | 0.1093 | 6.43E-05 | 0.0002 | 0.7300 |
| rs56188862 | 1 | 174189269 | C | T | 0.3862 | 1.57E-04 | 0.0001 | 0.1900 |
| rs72797284 | 5 | 152031650 | G | A | 0.2711 | 4.32E-05 | 0.0001 | 0.7400 |
| rs149805207 | 6 | 137095269 | G | A | 0.0086 | -5.17E-05 | 0.0007 | 0.9400 |
| rs10741694 | 11 | 16286183 | C | T | 0.6281 | -1.25E-04 | 0.0001 | 0.3000 |
| rs141071726 | 7 | 17558580 | A | G | 0.0270 | -4.34E-05 | 0.0004 | 0.9100 |
| rs1481012 | 4 | 89039082 | G | A | 0.1129 | -1.23E-04 | 0.0002 | 0.5000 |
| rs7757102 | 6 | 137222671 | G | A | 0.5551 | -2.94E-04 | 0.0001 | 0.0120 |
| rs977474 | 12 | 11284772 | T | C | 0.8336 | -2.31E-04 | 0.0002 | 0.1400 |
| rs6829 | 13 | 111531264 | T | C | 0.5956 | -4.51E-07 | 0.0001 | 0.9900 |
| rs2279844 | 17 | 40819809 | A | G | 0.3778 | 1.49E-04 | 0.0001 | 0.2100 |
| rs4808193 | 19 | 19410622 | C | T | 0.3346 | -2.70E-04 | 0.0001 | 0.0280 |
| rs10752269 | 10 | 12692902 | A | G | 0.5072 | -3.25E-05 | 0.0001 | 0.7800 |
| rs2645929 | 13 | 56444529 | G | A | 0.8130 | 1.87E-04 | 0.0001 | 0.2100 |

Abbreviation: SNP, single nucleotide polymorphism; Chr, chromosome; EAF, effect allele frequency; SE, standard error.

**Table S4: Mendelian randomization analysis of tea intake and lung cancer**

| SNP | Chr | position | A1 | A2 | EAF | Beta | SE | P-value |
| --- | --- | --- | --- | --- | --- | --- | --- | --- |
| rs57462170 | 3 | 50239803 | A | G | 0.1088 | -1.99E-04 | 0.0003 | 0.5200 |
| rs17685 | 7 | 75616105 | A | G | 0.2770 | 1.30E-04 | 0.0002 | 0.5500 |
| rs17576658 | 13 | 100272019 | A | G | 0.2473 | -4.89E-05 | 0.0002 | 0.8300 |
| rs2645929 | 13 | 56444529 | G | A | 0.8131 | 1.04E-04 | 0.0002 | 0.6800 |
| rs10764990 | 10 | 129152608 | A | G | 0.6072 | 1.45E-05 | 0.0002 | 0.9400 |
| rs4808193 | 19 | 19410622 | C | T | 0.3349 | 5.32E-04 | 0.0002 | 0.0099 |
| rs149805207 | 6 | 137095269 | G | A | 0.0085 | 7.37E-04 | 0.0012 | 0.5200 |
| rs7757102 | 6 | 137222671 | G | A | 0.5553 | 1.06E-04 | 0.0002 | 0.5900 |
| rs57631352 | 19 | 4338173 | G | A | 0.2963 | 1.52E-04 | 0.0002 | 0.4800 |
| rs2279844 | 17 | 40819809 | A | G | 0.3796 | -4.27E-04 | 0.0002 | 0.0330 |
| rs9624470 | 22 | 24820268 | A | G | 0.5801 | 2.55E-05 | 0.0002 | 0.9000 |
| rs4817505 | 21 | 34343828 | C | T | 0.3893 | -1.81E-04 | 0.0002 | 0.3600 |
| rs10741694 | 11 | 16286183 | C | T | 0.6277 | -2.93E-05 | 0.0002 | 0.8800 |
| rs1156588 | 2 | 58515375 | G | A | 0.2100 | -4.58E-04 | 0.0002 | 0.0550 |
| rs1481012 | 4 | 89039082 | G | A | 0.1125 | -7.06E-05 | 0.0003 | 0.8200 |
| rs72797284 | 5 | 152031650 | G | A | 0.2707 | 2.40E-05 | 0.0002 | 0.9100 |
| rs2351187 | 10 | 86850616 | A | G | 0.3189 | -2.98E-04 | 0.0002 | 0.1600 |
| rs10752269 | 10 | 12692902 | A | G | 0.5064 | -2.20E-04 | 0.0002 | 0.2600 |
| rs141071726 | 7 | 17558580 | A | G | 0.0266 | 1.86E-04 | 0.0006 | 0.7700 |
| rs9648476 | 7 | 39293033 | A | G | 0.6226 | 4.90E-05 | 0.0002 | 0.8100 |
| rs11587444 | 1 | 150722844 | G | A | 0.3942 | -2.28E-04 | 0.0002 | 0.2500 |
| rs6829 | 13 | 111531264 | T | C | 0.5961 | -1.79E-04 | 0.0002 | 0.3700 |
| rs17245213 | 11 | 1679769 | A | G | 0.2082 | -2.16E-04 | 0.0002 | 0.3700 |
| rs56188862 | 1 | 174189269 | C | T | 0.3886 | -2.29E-04 | 0.0002 | 0.2500 |
| rs2117137 | 3 | 89525505 | G | A | 0.4052 | -3.94E-05 | 0.0002 | 0.8400 |
| rs12591786 | 15 | 60902512 | T | C | 0.1591 | -2.01E-05 | 0.0003 | 0.9400 |

Abbreviation: SNP, single nucleotide polymorphism; Chr, chromosome; EAF, effect allele frequency; SE, standard error.

**Table S5: Mendelian randomization analysis of tea intake and COPD**

| SNP | Chr | position | A1 | A2 | EAF | Beta | SE | P-value |
| --- | --- | --- | --- | --- | --- | --- | --- | --- |
| rs56188862 | 1 | 174189269 | C | T | 0.3854 | -8.49E-05 | 0.0001 | 0.5663 |
| rs11587444 | 1 | 150722844 | G | A | 0.3919 | 3.50E-04 | 0.0001 | 0.0175 |
| rs1156588 | 2 | 58515375 | G | A | 0.2097 | 2.40E-04 | 0.0002 | 0.1749 |
| rs57462170 | 3 | 50239803 | A | G | 0.1092 | 1.67E-04 | 0.0002 | 0.4705 |
| rs2117137 | 3 | 89525505 | G | A | 0.4054 | -2.14E-04 | 0.0001 | 0.1435 |
| rs1481012 | 4 | 89039082 | G | A | 0.1124 | -2.82E-04 | 0.0002 | 0.2155 |
| rs34619 | 5 | 60465365 | A | G | 0.4319 | -2.52E-04 | 0.0001 | 0.0825 |
| rs72797284 | 5 | 152031650 | G | A | 0.2724 | -2.56E-04 | 0.0002 | 0.1136 |
| rs149805207 | 6 | 137095269 | G | A | 0.0085 | -1.75E-04 | 0.0008 | 0.8349 |
| rs17685 | 7 | 75616105 | A | G | 0.2783 | 8.85E-05 | 0.0002 | 0.5808 |
| rs141071726 | 7 | 17558580 | A | G | 0.0263 | 3.28E-05 | 0.0005 | 0.9435 |
| rs9648476 | 7 | 39293033 | A | G | 0.6245 | 1.76E-04 | 0.0001 | 0.2365 |
| rs13282783 | 8 | 22088975 | T | C | 0.2862 | -1.78E-04 | 0.0002 | 0.2659 |
| rs10752269 | 10 | 12692902 | A | G | 0.5077 | -1.81E-04 | 0.0001 | 0.2089 |
| rs10764990 | 10 | 129152608 | A | G | 0.6053 | -7.61E-05 | 0.0001 | 0.6047 |
| rs2351187 | 10 | 86850616 | A | G | 0.3221 | -1.29E-04 | 0.0002 | 0.4041 |
| rs10741694 | 11 | 16286183 | C | T | 0.6268 | -9.37E-05 | 0.0001 | 0.5295 |
| rs17245213 | 11 | 1679769 | A | G | 0.2071 | -9.66E-05 | 0.0002 | 0.5864 |
| rs977474 | 12 | 11284772 | T | C | 0.8341 | -1.56E-04 | 0.0002 | 0.4220 |
| rs17576658 | 13 | 100272019 | A | G | 0.2469 | 2.31E-05 | 0.0002 | 0.8900 |
| rs2645929 | 13 | 56444529 | G | A | 0.8126 | -8.24E-05 | 0.0002 | 0.6547 |
| rs6829 | 13 | 111531264 | T | C | 0.5964 | -9.51E-06 | 0.0001 | 0.9485 |
| rs12591786 | 15 | 60902512 | T | C | 0.1577 | 8.33E-05 | 0.0002 | 0.6775 |
| rs2279844 | 17 | 40819809 | A | G | 0.3765 | 1.14E-04 | 0.0001 | 0.4420 |
| rs57631352 | 19 | 4338173 | G | A | 0.2972 | 3.23E-04 | 0.0002 | 0.0405 |
| rs4808193 | 19 | 19410622 | C | T | 0.3349 | -1.82E-04 | 0.0002 | 0.2322 |
| rs4817505 | 21 | 34343828 | C | T | 0.3919 | 2.40E-04 | 0.0001 | 0.1043 |
| rs9624470 | 22 | 24820268 | A | G | 0.5803 | -8.43E-06 | 0.0001 | 0.9541 |

Abbreviation: SNP, single nucleotide polymorphism; Chr, chromosome; EAF, effect allele frequency; SE, standard error.

**Table S6: Mendelian randomization analysis of tea intake and acute bronchitis**

| SNP | Chr | position | A1 | A2 | EAF | Beta | SE | P-value |
| --- | --- | --- | --- | --- | --- | --- | --- | --- |
| rs7757102 | 6 | 137222671 | G | A | 0.5678 | -9.80E-03 | 0.0172 | 0.5672 |
| rs1481012 | 4 | 89039082 | G | A | 0.0745 | -1.10E-02 | 0.0325 | 0.7357 |
| rs17576658 | 13 | 100272019 | A | G | 0.2304 | 1.37E-02 | 0.0204 | 0.5004 |
| rs72797284 | 5 | 152031650 | G | A | 0.2091 | -6.20E-03 | 0.0209 | 0.7680 |
| rs4808193 | 19 | 19410622 | C | T | 0.2469 | -2.09E-02 | 0.0197 | 0.2899 |
| rs2279844 | 17 | 40819809 | A | G | 0.3651 | 3.10E-03 | 0.0176 | 0.8613 |
| rs10752269 | 10 | 12692902 | A | G | 0.6470 | -2.80E-03 | 0.0178 | 0.8732 |
| rs17245213 | 11 | 1679769 | A | G | 0.1742 | -2.57E-02 | 0.0224 | 0.2519 |
| rs13282783 | 8 | 22088975 | T | C | 0.4105 | 6.30E-03 | 0.0174 | 0.7154 |
| rs34619 | 5 | 60465365 | A | G | 0.4235 | -2.51E-02 | 0.0172 | 0.1448 |
| rs141071726 | 7 | 17558580 | A | G | 0.0143 | 1.95E-01 | 0.0731 | 0.0077 |
| rs56188862 | 1 | 174189269 | C | T | 0.3650 | -2.09E-02 | 0.0176 | 0.2359 |
| rs17685 | 7 | 75616105 | A | G | 0.3808 | 4.00E-03 | 0.0175 | 0.8169 |
| rs6829 | 13 | 111531264 | T | C | 0.5995 | 6.30E-03 | 0.0173 | 0.7163 |
| rs11587444 | 1 | 150722844 | G | A | 0.4036 | 5.10E-03 | 0.0173 | 0.7684 |
| rs977474 | 12 | 11284772 | T | C | 0.9158 | -6.85E-02 | 0.0306 | 0.0250 |
| rs12591786 | 15 | 60902512 | T | C | 0.0998 | 1.89E-02 | 0.0285 | 0.5064 |
| rs9624470 | 22 | 24820268 | A | G | 0.5476 | -1.43E-02 | 0.0171 | 0.4023 |
| rs149805207 | 6 | 137095269 | G | A | 0.0207 | -3.77E-02 | 0.0605 | 0.5332 |
| rs2351187 | 10 | 86850616 | A | G | 0.3140 | -4.55E-02 | 0.0183 | 0.0130 |
| rs9648476 | 7 | 39293033 | A | G | 0.6385 | 6.20E-03 | 0.0177 | 0.7255 |
| rs57462170 | 3 | 50239803 | A | G | 0.1415 | -5.24E-02 | 0.0245 | 0.0322 |
| rs1156588 | 2 | 58515375 | G | A | 0.2261 | -2.26E-02 | 0.0203 | 0.2663 |
| rs2645929 | 13 | 56444529 | G | A | 0.8496 | -5.20E-02 | 0.0238 | 0.0287 |
| rs10764990 | 10 | 129152608 | A | G | 0.5308 | 7.90E-03 | 0.0171 | 0.6451 |
| rs57631352 | 19 | 4338173 | G | A | 0.3355 | 5.60E-03 | 0.0180 | 0.7567 |
| rs10741694 | 11 | 16286183 | C | T | 0.7332 | 1.01E-02 | 0.0192 | 0.6004 |
| rs4817505 | 21 | 34343828 | C | T | 0.4327 | 1.40E-03 | 0.0172 | 0.9337 |
| Abbreviation: SNP, single nucleotide polymorphism; Chr, chromosome; EAF, effect allele frequency; SE, standard error | | | | | | | | |

**Table S7: Mendelian randomization analysis of tea intake and tuberculosis**

| SNP | Chr | position | A1 | A2 | EAF | Beta | SE | P-value |
| --- | --- | --- | --- | --- | --- | --- | --- | --- |
| rs9648476 | 7 | 39293033 | A | G | 0.6228 | -1.28E-04 | 0.0002 | 0.4000 |
| rs13282783 | 8 | 22088975 | T | C | 0.2860 | -7.39E-05 | 0.0002 | 0.6500 |
| rs2645929 | 13 | 56444529 | G | A | 0.8132 | -1.89E-04 | 0.0002 | 0.3100 |
| rs10752269 | 10 | 12692902 | A | G | 0.5063 | 4.62E-05 | 0.0001 | 0.7500 |
| rs2117137 | 3 | 89525505 | G | A | 0.4050 | 2.24E-04 | 0.0001 | 0.1300 |
| rs72797284 | 5 | 152031650 | G | A | 0.2708 | -2.55E-04 | 0.0002 | 0.1200 |
| rs11587444 | 1 | 150722844 | G | A | 0.3935 | -2.37E-05 | 0.0001 | 0.8700 |
| rs17245213 | 11 | 1679769 | A | G | 0.2081 | 2.56E-05 | 0.0002 | 0.8900 |
| rs17685 | 7 | 75616105 | A | G | 0.2774 | 2.32E-04 | 0.0002 | 0.1500 |
| rs977474 | 12 | 11284772 | T | C | 0.8336 | -2.18E-05 | 0.0002 | 0.9100 |
| rs17576658 | 13 | 100272019 | A | G | 0.2471 | -1.61E-04 | 0.0002 | 0.3400 |
| rs6829 | 13 | 111531264 | T | C | 0.5963 | 2.22E-04 | 0.0001 | 0.1400 |
| rs10764990 | 10 | 129152608 | A | G | 0.6074 | 2.08E-04 | 0.0001 | 0.1600 |
| rs12591786 | 15 | 60902512 | T | C | 0.1589 | -2.64E-04 | 0.0002 | 0.1900 |
| rs1156588 | 2 | 58515375 | G | A | 0.2101 | 3.89E-04 | 0.0002 | 0.0300 |
| rs7757102 | 6 | 137222671 | G | A | 0.5556 | -2.43E-04 | 0.0001 | 0.0970 |
| rs34619 | 5 | 60465365 | A | G | 0.4308 | -7.17E-05 | 0.0001 | 0.6300 |
| rs2279844 | 17 | 40819809 | A | G | 0.3796 | 2.62E-05 | 0.0001 | 0.8600 |
| rs2351187 | 10 | 86850616 | A | G | 0.3189 | 1.92E-04 | 0.0002 | 0.2200 |
| rs4808193 | 19 | 19410622 | C | T | 0.3352 | 1.74E-04 | 0.0002 | 0.2600 |
| rs4817505 | 21 | 34343828 | C | T | 0.3895 | -1.02E-04 | 0.0001 | 0.4900 |
| rs56188862 | 1 | 174189269 | C | T | 0.3878 | 1.14E-04 | 0.0001 | 0.4400 |
| rs57631352 | 19 | 4338173 | G | A | 0.2969 | -6.80E-05 | 0.0002 | 0.6700 |
| rs10741694 | 11 | 16286183 | C | T | 0.6277 | 1.25E-04 | 0.0002 | 0.4100 |
| rs9624470 | 22 | 24820268 | A | G | 0.5797 | 1.16E-05 | 0.0001 | 0.9400 |
| Abbreviation: SNP, single nucleotide polymorphism; Chr, chromosome; EAF, effect allele frequency; SE, standard error | | | | | | | | |

**Table S8: Mendelian randomization analysis of tea intake and pneumonia**

| SNP | Chr | position | A1 | A2 | EAF | Beta | SE | P-value |
| --- | --- | --- | --- | --- | --- | --- | --- | --- |
| rs2351187 | 10 | 86850616 | A | G | 0.3189 | -1.86E-02 | 0.0107 | 0.0807 |
| rs57631352 | 19 | 4338173 | G | A | 0.2969 | 3.97E-03 | 0.0109 | 0.7148 |
| rs17576658 | 13 | 100272019 | A | G | 0.2471 | -1.19E-02 | 0.0115 | 0.3004 |
| rs56188862 | 1 | 174189269 | C | T | 0.3878 | -3.61E-04 | 0.0102 | 0.9717 |
| rs7757102 | 6 | 137222671 | G | A | 0.5556 | 6.15E-03 | 0.0100 | 0.5377 |
| rs2117137 | 3 | 89525505 | G | A | 0.4050 | -9.69E-03 | 0.0101 | 0.3361 |
| rs57462170 | 3 | 50239803 | A | G | 0.1088 | 2.34E-03 | 0.0160 | 0.8834 |
| rs11587444 | 1 | 150722844 | G | A | 0.3935 | -2.90E-02 | 0.0102 | 0.0042 |
| rs10752269 | 10 | 12692902 | A | G | 0.5063 | 1.05E-02 | 0.0099 | 0.2904 |
| rs9648476 | 7 | 39293033 | A | G | 0.6227 | -1.43E-02 | 0.0102 | 0.1629 |
| rs10764990 | 10 | 129152608 | A | G | 0.6074 | 3.90E-03 | 0.0101 | 0.7010 |
| rs17245213 | 11 | 1679769 | A | G | 0.2081 | -2.22E-02 | 0.0122 | 0.0691 |
| rs12591786 | 15 | 60902512 | T | C | 0.1589 | 2.24E-02 | 0.0137 | 0.1032 |
| rs149805207 | 6 | 137095269 | G | A | 0.0086 | -6.07E-03 | 0.0587 | 0.9177 |
| rs6829 | 13 | 111531264 | T | C | 0.5963 | -1.13E-02 | 0.0101 | 0.2637 |
| rs1481012 | 4 | 89039082 | G | A | 0.1122 | -1.51E-02 | 0.0157 | 0.3375 |
| rs4817505 | 21 | 34343828 | C | T | 0.3895 | 2.13E-03 | 0.0102 | 0.8348 |
| rs2645929 | 13 | 56444529 | G | A | 0.8132 | 7.86E-03 | 0.0127 | 0.5368 |
| rs141071726 | 7 | 17558580 | A | G | 0.0267 | 2.52E-02 | 0.0319 | 0.4286 |
| rs4808193 | 19 | 19410622 | C | T | 0.3352 | -1.46E-02 | 0.0105 | 0.1637 |
| rs72797284 | 5 | 152031650 | G | A | 0.2708 | -7.57E-03 | 0.0111 | 0.4970 |
| rs2279844 | 17 | 40819809 | A | G | 0.3796 | 2.04E-02 | 0.0102 | 0.0459 |
| rs1156588 | 2 | 58515375 | G | A | 0.2100 | -3.32E-03 | 0.0122 | 0.7853 |
| rs9624470 | 22 | 24820268 | A | G | 0.5797 | -1.08E-02 | 0.0101 | 0.2827 |
| rs17685 | 7 | 75616105 | A | G | 0.2774 | -7.59E-04 | 0.0110 | 0.9452 |
| rs10741694 | 11 | 16286183 | C | T | 0.6278 | 2.91E-03 | 0.0103 | 0.7765 |

Abbreviation: SNP, single nucleotide polymorphism; Chr, chromosome; EAF, effect allele frequency; SE, standard error
